# A prospective case–control and molecular epidemiological study of human cases of Shiga toxin-producing *Escherichia coli* in New Zealand

**DOI:** 10.1186/1471-2334-13-450

**Published:** 2013-09-30

**Authors:** Patricia Jaros, Adrian L Cookson, Donald M Campbell, Thomas E Besser, Smriti Shringi, Graham F Mackereth, Esther Lim, Liza Lopez, Muriel Dufour, Jonathan C Marshall, Michael G Baker, Steve Hathaway, Deborah J Prattley, Nigel P French

**Affiliations:** 1 Molecular Epidemiology and Public Health Laboratory, Hopkirk Research Institute, Massey University, Private Bag, 11 222, Palmerston North 4442, New Zealand; 2 Animal Nutrition & Health Group, AgResearch Ltd, Grasslands Research Centre, Private Bag, 11 008, Palmerston North 4442, New Zealand; 3 Ministry for Primary Industries, PO Box 2526, Wellington 6140, New Zealand; 4 College of Veterinary Medicine, Veterinary Microbiology and Pathology, Washington State University, Pullman, WA, USA; 5 Institute of Environmental Science & Research Ltd, PO Box 40 158, Upper Hutt 5140, New Zealand; 6 Institute of Environmental Science & Research Ltd, PO Box 50 348, Porirua, Wellington 5240, New Zealand; 7 Department of Public Health, University of Otago, Wellington, PO Box 7343, Wellington 6242, New Zealand; 8 Allan Wilson Centre for Molecular Ecology & Evolution, Palmerston North, New Zealand

**Keywords:** Prospective case–control study, Sporadic STEC infections, New Zealand, Risk factors, Source attribution, Cattle, Molecular epidemiology, Pathways of infection, Population attributable fractions

## Abstract

**Background:**

Shiga toxin-producing *Escherichia coli* (STEC) O157:H7 and related non-O157 STEC strains are enteric pathogens of public health concern worldwide, causing life-threatening diseases. Cattle are considered the principal hosts and have been shown to be a source of infection for both foodborne and environmental outbreaks in humans. The aims of this study were to investigate risk factors associated with sporadic STEC infections in humans in New Zealand and to provide epidemiological information about the source and exposure pathways.

**Methods:**

During a national prospective case–control study from July 2011 to July 2012, any confirmed case of STEC infection notified to regional public health units, together with a random selection of controls intended to be representative of the national demography, were interviewed for risk factor evaluation. Isolates from each case were genotyped using pulsed-field gel electrophoresis (PFGE) and Shiga toxin-encoding bacteriophage insertion (SBI) typing.

**Results:**

Questionnaire data from 113 eligible cases and 506 controls were analysed using multivariate logistic regression. Statistically significant animal and environmental risk factors for human STEC infections were identified, notably 'Cattle livestock present in meshblock’ (the smallest geographical unit) (odds ratio 1.89, 95% CI 1.04–3.42), 'Contact with animal manure’ (OR 2.09, 95% CI 1.12–3.90), and 'Contact with recreational waters’ (OR 2.95, 95% CI 1.30–6.70). No food-associated risk factors were identified as sources of STEC infection. *E. coli* O157:H7 caused 100/113 (88.5%) of clinical STEC infections in this study, and 97/100 isolates were available for molecular analysis. PFGE profiles of isolates revealed three distinctive clusters of genotypes, and these were strongly correlated with SBI type. The variable 'Island of residence’ (North or South Island of New Zealand) was significantly associated with PFGE genotype (*p* = 0.012).

**Conclusions:**

Our findings implicate environmental and animal contact, but not food, as significant exposure pathways for sporadic STEC infections in humans in New Zealand. Risk factors associated with beef and dairy cattle suggest that ruminants are the most important sources of STEC infection. Notably, outbreaks of STEC infections are rare in New Zealand and this further suggests that food is not a significant exposure pathway.

## Background

Shiga toxin-producing *Escherichia coli* (STEC) O157:H7 and related non-O157 STEC strains are pathogens of public health concern worldwide. They can cause severe outbreaks of gastrointestinal illness with clinical symptoms ranging from diarrhoea and haemorrhagic colitis to the life-threatening haemolytic uraemic syndrome [1]. Ruminants, particularly cattle, are considered to be an important reservoir of STEC, shedding the pathogen via faeces [2-4], and are a primary source of foodborne and environmental outbreaks of STEC in humans [5,6].

Food products of animal and plant origin have been confirmed as vehicles of disease transmission in case–control studies of STEC outbreaks and sporadic STEC infections; these included raw milk [7-9], unpasteurised cheese [10], undercooked hamburgers [11-13], sausages [14,15], leafy lettuce [16] and unpasteurised apple cider [17]. Implicated food vehicles were most commonly contaminated directly or indirectly with ruminant faeces containing STEC before or after processing. Similarly, faecally contaminated recreational waters and water supplies have been identified as environmental sources of human STEC infections [18-20]. Exposures to farming environments have been reported as risk factors of sporadic STEC infections, particularly for young children [8,21].

Improvements in surveillance of STEC infections, following their recognition as serious public health concerns, have resulted in an overall increasing trend of STEC notifications at the international level. Over the past decade, non-O157 STEC cases have been reported more frequently in the USA and the EU [22,23], a feature that might be attributed to improved laboratory methods for isolation of non-O157 STEC serotypes and additional laboratory testing of specimens for non-O157 STECs.

Since 1993, when New Zealand’s first case of STEC infection in humans was reported [24], the annual number of notified STEC cases has increased steadily. In 2012, 147 cases of STEC (3.3 cases per 100,000 population) were recorded in the national surveillance database (EpiSurv) used by regional public health units (PHU) to record epidemiological data from notified cases of communicable and other diseases [25]. All but five cases were confirmed by culture isolation and identified as STEC serotypes O157:H7 (83.8%) and non-O157 (16.2%). Although the majority of reported STEC cases in New Zealand are caused by serotype O157:H7, the percentage of non-O157 STEC cases has increased steadily over the past five years from 1.6% in 2008 to 16.2% in 2012.

While STEC infections in New Zealand appear as sporadic cases or small clusters, little is known about the relative importance of cattle as a reservoir, or the relative contribution of different exposure pathways to human cases of STEC.

The primary objective of this study was the identification of risk factors associated with sporadic STEC infections acquired in New Zealand so as to gain epidemiological knowledge on the source and exposure pathways for this disease. A second objective was to conduct a molecular epidemiological investigation of STEC isolates from clinical cases.

## Methods

### Study design, definition of cases and controls

A national prospective case–control study was conducted in New Zealand from 18^th^ July 2011 to 31^st^ July 2012. A case was defined as a patient with (i) clinical symptoms of diarrhoea and/or haemolytic uraemic syndrome and/or thrombotic thrombocytopaenia purpura, (ii) an onset of clinical disease at a maximum of two weeks prior to being reported to a PHU, (iii) an infection most likely acquired in New Zealand, (iv) confirmed by isolation of STEC from a clinical specimen, and (v) the primary STEC infection in a household. Study cases were interviewed by phone or in person by trained PHU staff using a questionnaire on multiple risk factors potentially associated with STEC infections.

Study controls, intended to be representative of the national demography, were selected randomly from the New Zealand population. An eligible control had to be free of symptoms of diarrhoea or any other gastrointestinal disease at the time of interview, and in the two weeks prior to the interview. Monthly quotas of controls were recruited by a professional survey provider (UMR Research, Wellington, New Zealand) using random landline dialling from the New Zealand phone directory. Controls were contacted in the third week of every month. In each household, the individual with the last birthday was chosen as the study participant. A computer-assisted telephone interview was conducted by a trained team of assigned interviewers using the same questionnaire as that used for cases.

Informed consent was obtained from all study participants before being interviewed. For study cases and controls aged <18 years, a parent or adult caregiver served as the interview respondent after their consent was acquired.

### Questionnaire

A standardised questionnaire (Additional file 1) was used to collect data from study cases and controls concerning potential risk factors for infection in the two weeks before onset of disease (cases) and the telephone interview (controls). The questionnaire covered demographic characteristics and exposure categories such as food consumed (treated/raw milk and products thereof, various raw/pink meats, fish, raw fruit and vegetables, and purchased fruit juices), dining locations, supply of drinking water (town supply, private bore, roof run-off, creek, tanker truck), contact with recreational waters, hunting activities, contacts with animals and humans, recent travels, and medications taken (antibiotic and antacid). To investigate the spatial distribution of study participants, while protecting privacy, cases and controls were asked to name the nearest school to their home to assign their geographical locality. The month and year of interview was recorded to investigate the seasonality of disease. The questionnaire was cognitive and pilot tested.

### Sample sizes of cases and controls

Epi Info™ software [26] was used to calculate the sample size for cases and to perform power calculations for three different expected frequencies of exposure among controls. Based on a predicted sample size of 150–170 cases (expected number of cases based on STEC cases notified nationally in two preceding years) and an attempted case to control ratio of 1:3, there was sufficient statistical power (at least 80%) at a confidence level of 95% to detect an odds ratio of 3.0, using 5%, 20% and 80% as the expected frequencies of exposure among controls. Hence, the sample size of controls was set at 506 and included oversampling of children 0–4 years of age (*n* = 200) to provide a similar predicted ratio of cases and controls (1:3) as this age group showed the highest number of reported STEC cases in the past. A monthly quota of 42 controls was interviewed by the survey provider.

### STEC isolates of study cases

Clinical cases were confirmed by culture isolation of STEC from clinical specimens submitted to medical laboratories or the Enteric Reference Laboratory (ERL, Institute of Environmental Science & Research Ltd, Upper Hutt, New Zealand). STEC isolates were submitted to ERL for serotyping, testing for the presence of virulence genes (*ehx*A, *eae*, *stx*1, *stx*2), and genotyping using pulsed-field gel electrophoresis (PFGE, restriction enzyme *Xba*I). Isolates were sent to the Molecular Epidemiology and Public Health Laboratory (^*m*^EpiLab, Hopkirk Research Institute, Massey University, Palmerston North, New Zealand) for screening for the presence of virulence gene subtype *stx*2c. In addition, *E. coli* O157:H7 isolates were genotyped using Shiga toxin (Stx)-encoding bacteriophage insertion (SBI) typing (Prof. Thomas E. Besser and colleagues at Washington State University, Pullman, USA) [27,28].

### Ethical approval

This study was approved by the Multi-region Ethics Committee, Wellington, New Zealand, on 17 June 2011; reference number MEC/11/04/043.

### Data management and statistical analysis

R software (version 2.15.2) [29] was used for all statistical analysis, with significance set at *p* <0.05.

Datasets of cases and controls were screened for completeness prior to analysis. Descriptive statistics were calculated for each study group. To account for potential confounding from imperfect frequency matching on age, the variable 'Age’ was categorised by grouping 'pre-school children’ (0–4 years), 'children/students’ (5–19 years), and 'adults’ (>19 years).

To illustrate the spatial distribution of study participants, New Zealand Transverse Mercator coordinates (NZTM2000) of named schools were plotted, using R packages 'maptools’ [30] and 'spatstat’ [31]. Based on the spatial distribution of cases and controls, a relative risk surface of STEC cases for New Zealand was produced, using R package 'sparr’ [32]. To account for spatial heterogeneity, an adaptive estimate was utilised for case and control densities with an average smoothing bandwidth of 50 km. Areas with values >0.0 indicate increased relative risks of STEC infection. For comparison, cattle densities were mapped by regions of New Zealand; using the sum of beef and dairy cattle numbers from 2011 [33] divided by the area (km^2^) of each region.

In addition to the data generated by the case–control questionnaires, information on ruminant livestock numbers from a national livestock database [34] was used in two separate analyses. Firstly, additional variables were generated, representing whether particular species of livestock (dairy cattle, beef cattle, sheep, and deer) were farmed in meshblocks (the smallest geographic unit of statistical data collected for Statistics New Zealand) in which the cases and controls resided. These additional variables (presence/absence, numbers and density of each species) were used in the logistic regression analysis of the case–control dataset.

Secondly, in order to extend the analysis of the relationship between ruminant livestock (dairy cattle, beef cattle, sheep, and deer) and the risk of STEC, a separate logistic regression analysis was conducted at the meshblock level. The relationship between ruminant livestock (presence/absence, numbers and density of each species) and the risk of STEC notification in all meshblocks of New Zealand was assessed. In essence, this analysis used the cases from the case–control study, but extended the control set to consider the entire population of New Zealand.

For both logistic regression analyses, ruminant livestock data from 2009 were used as they represented the most reliable recent data that could be linked to geographical boundaries (meshblocks) and the most recent human census data. The last population census (2006) estimated a national human population of 4,027,527.

### Multivariate logistic regression model building

Questionnaire answers of “unknown” or “not sure” were treated as missing values of the exposure variables. Exposure variables were analysed using univariate and multivariate logistic regression to identify risk factors associated with sporadic STEC cases. Exposure variables with Wald test or Likelihood ratio tests *p*-values <0.20 in univariate analysis were tested for correlation, and included in an initial multivariate model if their correlation values were < ±0.30.

To generate a preliminary multivariate model, stepwise backward- and forward-elimination of least significant variables and those with correlation values of ≥ ±0.30, respectively, was used, while eliminated variables were assessed for confounding. The confounding effect was determined by a change of >30% in a variable coefficient in the model after another variable was dropped from or added to the model. Variables which demonstrated confounding were retained in the model even if they were non-significant. Biologically plausible interactions between variables were assessed to generate the final multivariate model.

To adjust for a proportion of missing values in relevant variables such as 'Contact with animal manure’ (cases: *n* = 13, controls: *n* = 7), 'Contact with children wearing nappies’ (cases: *n* = 6, controls: *n* = 4), and 'Contact with person vomiting/having gastrointestinal disease’ (cases: *n* = 10, controls: *n* = 16), multiple imputations by chained equations [35] were applied on the final multivariate model using R package 'mice’ [36]. Likelihood ratio tests and the le Cessie-van Houwelingen normal test statistics [37] were applied to evaluate the model’s significance and goodness-of-fit, respectively, using R package 'rms’ [38]. Models were compared using the Akaike information criterion (AIC), a measure of the relative goodness of fit.

For the extended analysis of the relationship between ruminant livestock and the risk of STEC at the meshblock level, a second multivariate logistic regression model was built. In this analysis the number of STEC cases out of the population in each meshblock was the outcome variable (a two-column vector of the number of cases out of the population in each meshblock) and variables representing each ruminant species per meshblock were considered as exposure variables (presence/absence, numbers and density).

### Population attributable fractions (PAF)

To assess the proportion of sporadic STEC disease in the study population attributable to a specific exposure, the variable’s population attributable fraction (PAF_i_) was computed. PAF of variables associated with increased risk of STEC infection were estimated using the following formula [39,40]:

$$\mathrm{PA}F_{i}=\frac{p_{i}\left(\mathrm{aO}R_{i}-1\right)}{\mathrm{aO}R_{i}}\times 100\%,$$

where *p*_*i*_ is the proportion of all study cases within a categorical variable and a reference category denoted by *i* = 1, and *aOR*_*i*_ is the adjusted variable-specific odds ratio derived from the final multivariate model. Medians and 95% credible intervals were computed from 1,000 simulations as described in Stafford *et al*. [39].

### Molecular analysis of *E. coli* O157:H7 isolates

PFGE profiles of the clinical *E. coli* O157:H7 isolates were analysed and compared using BioNumerics software (version 6.6) [41] to create a dendrogram applying UPGMA (unweighted pair group method with arithmetic mean) cluster analysis using the Dice similarity coefficient, with a band matching tolerance of 1%.

Fisher’s exact test was used to evaluate the association between SBI genotypes and exposure variables considered in the multivariate logistic regression analysis. A distance matrix of isolates’ PFGE profiles was generated in BioNumerics and linked with exposure variables, to analyse the molecular relatedness of isolates and estimate the proportional contribution of these variables to the molecular variation. Multidimensional scaling plots and permutational multivariate analysis of variance (PERMANOVA+, version 1.0.4) were used for this analysis using Primer 6 (version 6.1.14) [42].

## Results

### Study population, spatial and temporal epidemiology

A total of 123 STEC cases meeting the case definition were notified to PHUs during the study period. Eight cases refused to participate in the study, thus resulting in a 93.5% response rate. Two potential cases were excluded, one due to a high probability of having acquired the infection overseas and the other due to severe illness. Therefore, 113 STEC cases were included in this study, of which 75 (66.4%) were interviewed by phone and 35 (31.0%) were visited by PHU staff; the interviewing methods of three cases were unknown. The majority of STEC cases (83.2%, 94/113) were interviewed within 0–5 days of notification (12 cases within 6–9 days, four cases within 10–12 days, and three cases within 21 days). Of these 113 STEC cases, 100 (88.5%) were *E. coli* O157:H7 and 13 (11.5%) were non-O157 STEC.

To recruit 506 controls, including 200 0–4 year-old children, a total of 7864 phone calls were made. Contact was established for 66.8% (5254/7864) of phone calls and of those contacted, 62.2% (3266/5254) were interested in participating in the study (response rate). From 3266 interested respondents, 84.5% (2760/3266) were not eligible mainly because of not fitting the required monthly quota of 0–4 year-olds (93.6% (2583/2760)), or not meeting the selection criteria.

**Figure 1:**
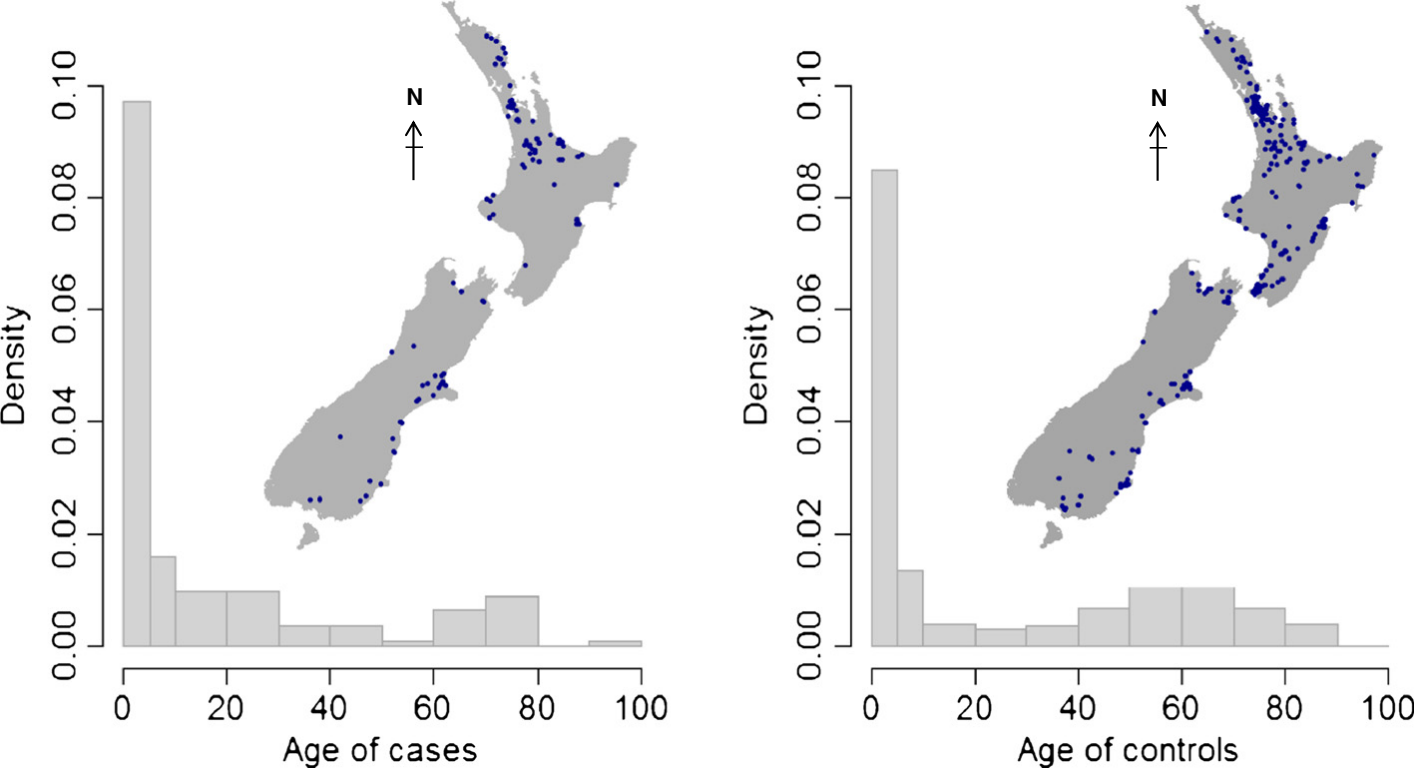
**Age and spatial distribution of STEC cases (*n*** **= 113) and controls (*n*** **= 506) across New Zealand.**

Males comprised 52.2% (59/113) of cases and 42.9% (217/506) of controls. The median age of cases and controls were 7.0 years (interquartile range 2.0–29.0) and 11.5 years (IQR 3.0–58.0), respectively. The age and spatial distribution of cases and controls are shown in Figure 1.

The proportional distributions of participants stratified by age categories were: 46.0% cases (52/113) and 40.3% controls (204/506) for 0–4 year-old pre-school children; 20.4% cases (23/113) and 12.1% controls (61/506) for 5–19 year-old children/students; and 33.6% cases (38/113) and 47.6% controls (241/506) for >19 year-old adults.

**Figure 2:**
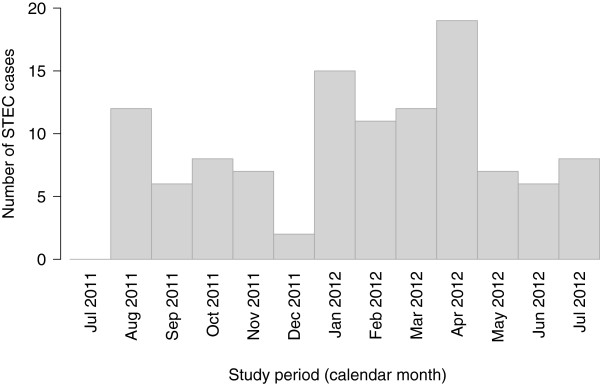
**Temporal distribution of sporadic STEC cases (*n*** **= 113) from July 2011 to July 2012.**

The temporal distribution of cases during the study period showed a peak in summer/autumn (January until April), with no cases reported in July 2011 (Figure 2).

**Figure 3:**
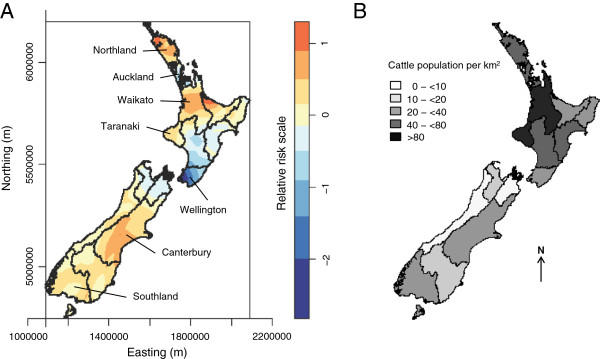
**(A) Relative risk estimates of sporadic STEC infection across New Zealand and (B) cattle density from 2011. (A)** The bivariate kernel density plot shows estimates of relative risks of STEC infection across regions in New Zealand. Values >0 on the relative risk scale indicate increased risk of infection. **(B)** Total cattle density (dairy and beef animals/km^2^) from 2011 is shown to indicate areas with high cattle densities. Similar plots of the densities of dairy, beef, sheep, and deer are provided in the supplementary material (Additional file 2).

Based on the spatial distribution of cases and controls across New Zealand (Figure 1), increased relative risk estimates of STEC infections were observed in regions such as Northland, Waikato, Taranaki, Canterbury, and Southland, while reduced risks were found in high density urban areas in the Auckland and Wellington regions (Figure 3A). For comparison, areas with high ruminant livestock densities are shown in Figure 3B and Additional file 2.

### Risk factors

Bivariate logistic regression results (adjusted for age categories) are provided in the supplementary material (Additional file 3). Statistically significant risk factors and confounding variables ('Eating seafood’, 'Dining outside home’, 'Water supply to home from private bore/spring/creek/or stream’, 'Contact with children wearing nappies’, and 'Taking antacids’) of the final multivariate logistic regression model with imputations are presented in Table 1. The equivalent final model without imputation is provided in the supplementary material (Additional file 4).

**Table 1:** Multivariate logistic regression model showing risk factors for sporadic cases of STEC infections in New Zealand

**Variable**		**Coefficient (SE)**^**a**^	**Adjusted odds ratio (95% CI)**^**b**^	***p*-value**^**c**^
Other household member having contact with animals other than household pets^d^*				
for 0–4 year-old		1.39 (0.42)	4.03 (1.78–9.13)	0.001
for 5–19 year-old		-0.77 (0.67)	0.47 (0.13–1.72)	0.251
for >19 year-old		0.30 (0.49)	1.35 (0.51–3.56)	0.541
Cattle livestock present in meshblock		0.64 (0.30)	1.89 (1.04–3.42)	0.037
Contact with animal manure		0.74 (0.32)	2.09 (1.12–3.90)	0.021
Contact with recreational waters		1.08 (0.42)	2.95 (1.30–6.70)	0.010
Travelled to areas in New Zealand with interrupted or no main water supply		0.89 (0.43)	2.43 (1.04–5.65)	0.040
Handling raw offal		-0.94 (0.35)	0.39 (0.20–0.78)	0.008
Drinking refrigerated fruit juice from supermarket		-1.37 (0.32)	0.25 (0.14–0.47)	<0.001
Visiting childcare/kindergarten/or school		-0.92 (0.31)	0.40 (0.22–0.73)	0.003
Eating raw vegetables		-0.65 (0.33)	0.52 (0.27–0.99)	0.046
Eating seafood		-0.52 (0.27)	0.59 (0.35–1.02)	0.057
Dining outside home		-0.44 (0.29)	0.65 (0.37–1.14)	0.133
Water supply to home from private bore/spring/creek/or stream		0.62 (0.39)	1.85 (0.86–4.00)	0.117
Contact with children wearing nappies		-0.33 (0.31)	0.72 (0.39–1.33)	0.298
Taking antacids		-0.84 (0.58)	0.43 (0.14–1.34)	0.147

Likelihood ratio test = 153.70 (df = 18, *p* < 0.001).

^a^Standard error.

^b^95% confidence interval.

^c^*p*-values were computed based on 50 imputations.

^d^This variable was modelled using a multiplicative interaction term comprising the variables 'Other household member having contact with animals other than household pets’ and 'Age’.

It can be interpreted as follows: a child 0–4 years of age is at significantly higher risk of being an STEC case, if another household member had contact with animals other than household pets, compared to a child of the same age without this risk factor.

*p-value = 0.064 for the variable 'Other household member having contact with animals other than household pets’ without the interaction term.

Animal and environmental exposures were identified as risk factors for sporadic STEC infections including; 'Other household member having contact with animals other than household pets’ for pre-school children aged 0–4, 'Cattle livestock present in meshblock’, 'Contact with animal manure’, 'Contact with recreational waters’, and 'Travelled to areas in New Zealand with interrupted or no main water supply’. Food items such as 'Drinking refrigerated fruit juice from supermarket’ and 'Eating raw vegetables’ were identified as having a protective effect rather than being risk factors for STEC infections. When the final multivariate logistic regression model was applied to *E. coli* O157:H7 cases only, the strength of associations and significance of variables remained relatively unchanged (data not shown), except for the variable 'Contact with recreational waters’, which became non-significant (adjusted odds ratio 2.13, 95% CI 0.84–5.42, *p* = 0.112). This could be explained by a higher proportion of non-O157 cases (30.8%, 4/13) being exposed to this risk factor compared to O157 cases (11.0%, 11/100).

For the multivariate analysis considering cases and the whole population at the meshblock level, the univariate logistic model identified significant associations between STEC and dairy cattle, beef cattle, and sheep, where variables with different functional forms were considered including presence/absence, numbers of animals and densities per km^2^. The best fitting variables, in terms of AIC, were presence/absence of cattle and sheep. These included presence of beef cattle (odds ratio 2.45, 95% CI 1.65–3.59, Wald test *p*-value <0.001, AIC = 1556.9), presence of dairy cattle (OR 2.14, 95% CI 1.27–3.42, *p* = 0.003, AIC = 1567.5), presence of all cattle (OR 2.40, 95% CI 1.62–3.52, *p* <0.001, AIC = 1557.6), and presence of sheep (OR 1.98, 95% CI 1.29–2.97, *p* = 0.001, AIC = 1565.7). When considered in multivariate models, there was strong confounding and collinearity between these variables but only species combinations of beef cattle with dairy cattle (Likelihood ratio test *p*-value <0.001, AIC = 1558.9), and dairy cattle with sheep (*p* = 0.005, AIC = 1566.6) provided biologically meaningful results. According to lowest AIC, the variable presence of beef cattle fitted the best.

### Population attributable fractions (PAF)

PAF of exposure variables associated with increased risk for sporadic STEC infections (Table 1) are summarised in Table 2. The interaction term 'Other household member having contact with animals other than household pets’ for 0–4 year-old children, 'Cattle livestock present in meshblock’ and 'Contact with animal manure’ showed the highest estimated proportions that could be attributed to STEC infections in the study population.

**Table 2:** Population attributable fractions (PAF in %) with 95% credible intervals (CrI in %) of identified risk factors

**Variable**		**Cases (n)**	**Proportion of cases (p)**	**Adjusted odds ratio**^**a**^	**PAF (95% CrI)**^**b**^
For children 0–4 years old: Other household member having contact with animals other than household pets					
No		28	0.549	Ref	-
Yes		23	0.451	4.03	16.82 (9.0–23.7)
Cattle livestock present in meshblock					
No		74	0.655	Ref	-
Yes		39	0.345	1.89	18.20 (0.6–29.4)
Contact with animal manure					
No		66	0.660	Ref	-
Yes		34	0.340	2.09	17.47 (4.4–27.7)
Contact with recreational waters					
No		97	0.866	Ref	-
Yes		15	0.134	2.95	9.41 (2.7–16.5)
Travelled to areas in NZ with interrupted or no main water supply					
No		96	0.865	Ref	-
Yes		15	0.135	2.43	8.17 (0.7–15.7)
Water supply to home from private bore/spring/creek/or stream					
No		89	0.802	Ref	-
Yes		22	0.198	1.85	9.46 (-2.5–18.8)

^a^Adjusted odds ratios were derived from multivariate logistic regression analysis model using multiple imputations by chained equations (Table 1).

^b^PAF and 95% CrI were computed based on 1000 simulations.

Ref = reference level for comparison.

### Molecular analysis of *E. coli* O157:H7 isolates

*E. coli* O157:H7 and non-O157 STECs, as confirmed by isolation, caused 100/113 (88.5%) and 13/113 (11.5%) of the STEC infections, respectively. The non-O157 STECs were of serogroups O26, O84, O103, O123, O176, O180, and ONT (O serogroup not typable). Only 97/100 O157:H7 isolates and their PFGE profiles were available for molecular analysis; PFGE profiles of non-O157 STEC isolates were not available. The most frequent SBI types of *E. coli* O157:H7 isolates were 1 (55/97, 56.7%), 3 (17/97, 17.5%), and 5 (20/97, 20.6%); equivalent to SBI genotypes AY2, WY12, and ASY2c/ASWY2c/SY2c, respectively, according to the recently proposed coding system by Shringi *et al*. [27]. All isolates of SBI type 1 (AY2) carried the *stx*2a gene, while all SBI type 3 (WY12) had both the *stx*2a and *stx*1 genes; all SBI type 5 (ASY2c/SY2c) contained only the *stx*2c gene.

**Figure 4:**
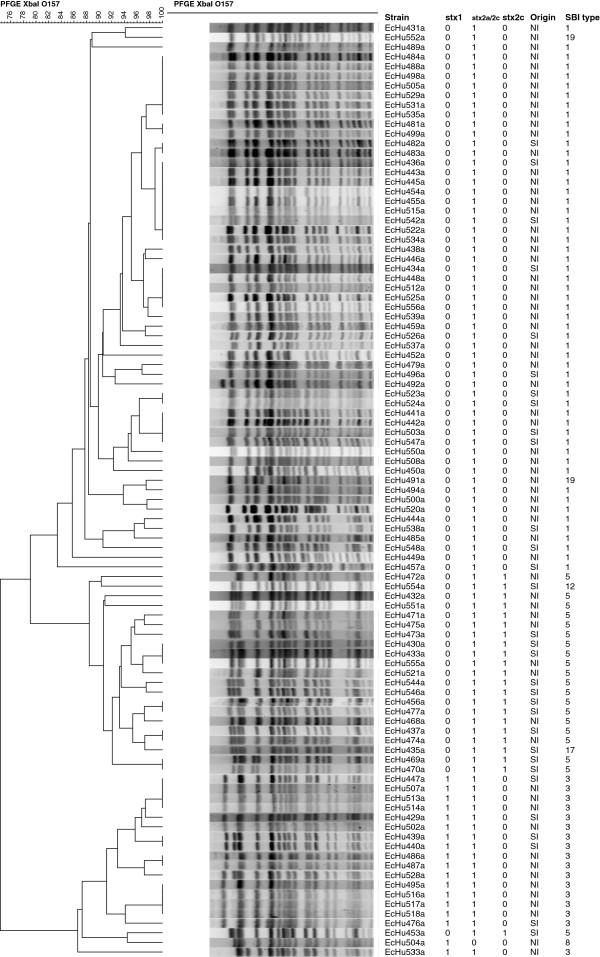
**Comparison of PFGE profiles from 97 human *E. coli *O157:H7 isolates.** PFGE profile comparison performed using UPGMA cluster analysis, Dice similarity coefficient, and 1% band matching tolerance. *stx*1, *stx*2a/c, *stx*2c virulence genes encoding for Shiga toxins present (1) or absent (0). Island of residence (Origin) presented as North Island (NI) or South Island (SI) of New Zealand, and genotypes of isolates as Shiga toxin (Stx)-encoding bacteriophage insertion (SBI) types.

PFGE profiles of the 97 human *E. coli* O157:H7 isolates were compared (Figure 4). The two small clusters of indistinguishable PFGE profiles (two clusters of seven and eight isolates) were not concurrent in space and time and therefore do not present clusters of infections or small outbreaks.

Four statistically significant relationships were observed between SBI types and exposure variables considered in the multivariate logistic regression analysis of the case–control study. These were SBI type vs. 'Age’ with SBI type 5 isolates being overrepresented in 0–4 year-old children (Fisher’s exact test, *p* = 0.009); SBI type vs. 'Island of residence’ with SBI type 5 isolates being associated with the South Island (*p* = 0.017); SBI type vs. 'Season’ with SBI type 3 isolates being overrepresented in autumn (*p* = 0.034), and SBI type vs. 'Contact with animal manure’ with SBI type 3 isolates being associated with direct exposure to animal manure (*p* = 0.047).

**Figure 5:**
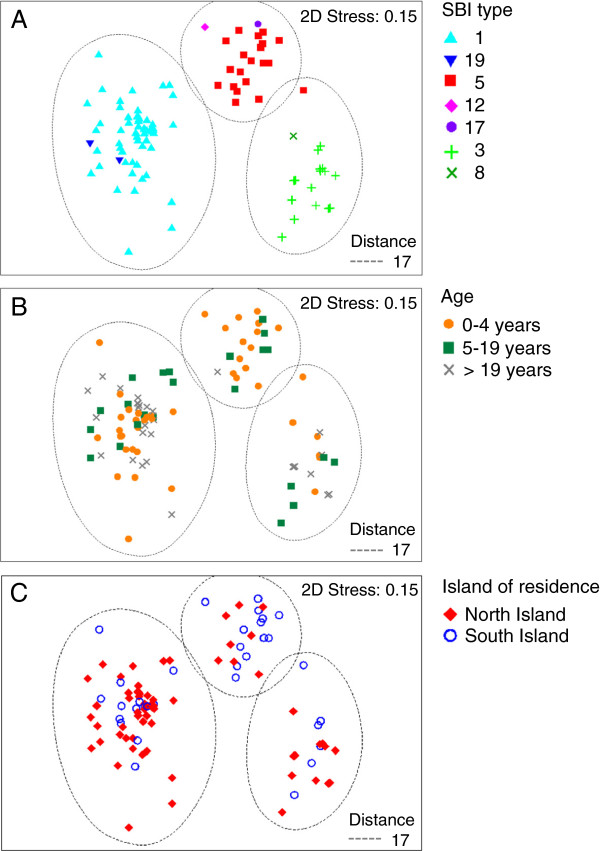
**Multidimensional scaling plots (MDS).** MDS showing the genotypic clustering of *E. coli* O157:H7 human isolates and **(A)** Shiga toxin-encoding bacteriophage insertion (SBI) types; **(B)** age categories; and **(C)** island of residence. The three clusters of isolates share a genetic difference of 17% (Distance = 17) based on the isolates’ PFGE profiles.

The molecular relatedness between PFGE profiles of *E. coli* O157:H7 isolates considering SBI types, age of cases and island of residence is shown in Figure 5. PFGE profile clusters were strongly associated with SBI types 1, 3, and 5 (Figure 5A). The cluster containing SBI type 5 was more prevalent in pre-school children (0–4 years) (Figure 5B) and in the South Island (Figure 5C), while SBI types 1 and 3 were found more frequently in the North Island (Figure 5C).

PERMANOVA analysis results in Table 3 show the proportional contribution of variables to the molecular variation of PFGE profiles of the *E. coli* O157:H7 isolates. Other than SBI type, only island of residence explained a significant amount of the variation in PFGE profiles in multivariate models.

**Table 3:** **PERMANOVA analysis of *E. coli *O157:H7 isolates**

**Variable**	**Df**^**a**^	**Mean square**	***p*-value**	**Perms**^**b**^	**Estimated component of variation (%)**
Island of residence	1	859.0	0.012	999	22.3
Residuals	95	187.5			77.7

^a^Degrees of freedom.

^b^Number of permutations.

## Discussion

This study was designed to identify risk factors associated with domestically-acquired sporadic STEC infections in humans in New Zealand. The results strongly suggest that direct exposure to animal and/or environmental sources of infection, most likely originating from dairy and beef livestock, is the most important contributor to the burden of sporadic STEC cases observed in New Zealand. No food items were identified as risk factors for sporadic STEC cases in this study.

### New Zealand–an agricultural country

To interpret our findings in context, it is essential to recognise that agriculture is New Zealand’s largest primary industry sector contributing to approximately 48% of New Zealand’s export earnings in 2009 [43]. In 2011, 6.1 million dairy cattle, 3.8 million beef cattle, 31.1 million sheep, and 1.0 million deer were recorded in New Zealand. By contrast the estimated human population was approximately 4.4 million with 14% living in rural areas and only about 1.2% working in the agricultural industry [33]. Pastoral agriculture is the predominant land use in New Zealand with dairy cattle farming in the flatter and/or wetter areas in Northland, Waikato, Taranaki, and Manawatu in the North Island; and Canterbury, West Coast, Otago, and Southland in the South Island; while sheep and beef cattle farming are practiced in hill and high country areas across both islands.

### Spatial and temporal epidemiology

The highest number of reported STEC infections in this study was in the youngest age category (children aged 0–4 years), which is consistent with New Zealand’s health surveillance reports [44-46], and the number of cases peaked in summer/autumn (January until April) [47].

The seasonality of cases is likely to be associated with environmental exposure during the warmer season, such as increased outdoor activities in recreational waters potentially contaminated with STEC from ruminant livestock, but could also be related to the seasonal variation in the prevalence of faecal shedding of STEC in cattle. These phenomena were observed in The Netherlands [48], Great Britain [49], and also in New Zealand during a recent two-year cross-sectional study conducted at four slaughter plants across the country [50]. Similarly, a recent multinational systematic review of seasonality in human zoonotic enteric diseases [51] confirmed a strong summer peak for STEC incidence.

The spatial distribution of sporadic STEC cases across New Zealand has suggested that infections might be associated with farming [45,46,52]. We observed increased relative risks of STEC infections in dairy farming regions (Northland, Waikato, Taranaki, Canterbury, and Southland), however, it was not possible to consider dairy and beef farming separately in the analysis of the case–control data due to the strong collinearity between these two variables. As all dairy farms in the meshblocks occupied by cases and controls also had beef cattle, hence the two variables were combined into a single variable comprising all cattle. The best fitting variable in the second analysis, which considered the entire population at the meshblock level, was the presence of beef cattle in the meshblock. Associations between STEC infections and areas with higher densities of cattle have been observed in previous studies conducted in The Netherlands [48], Finland [53], Scotland [54], Sweden [55], and Canada [56,57], providing evidence of direct or indirect contact with cattle as a likely source of infection.

### Risk factors

Since reporting of STEC commenced in New Zealand in 1997, cases have occurred sporadically or as small clusters throughout the country, suggesting highly dispersed animal and/or environmental exposures rather than STEC-contaminated food as likely sources of infection. In this study, animal and environmental contacts were identified as significant risk factors for sporadic STEC infections. A child 0–4 years of age was at significantly higher risk, if another household member had contact with animals other than household pets, compared to a child of the same age without this risk factor. This finding is biologically plausible and is more likely to occur in rural than urban settings; for example, when household members working on a farm could be a source of infection. Pre-school children might be exposed to contaminated work clothing and footwear harbouring pathogenic organisms. For example, *Campylobacter* was recovered from loose debris shaken off protective overalls worn on broiler farms [58]. Household occupation contact with farm animals (sheep or lambs) was also the major risk factor for *Salmonella* Brandenburg infection in a previous New Zealand case–control study where the infection also particularly affected rural children [59].

In addition, infants and pre-school children exhibit high frequencies of hand-to-mouth and object-to-mouth behaviour indoors and outdoors and this will inevitably increase the risk of ingesting pathogens from clothing, surfaces, objects, hands, and soil [60,61]. This is particularly apparent when children are raised in a farming environment, where they are more likely to be exposed to zoonotic and soil-associated pathogens than in urban areas. Therefore, practicing good hand-hygiene and supervising activities of infants and toddlers could help to reduce the risk of ingesting pathogens.

Although 'Living in a rural area’ was not statistically significant in our model, there was sufficient evidence that risk factors related to agricultural or rural characteristics were associated with sporadic STEC infection, and these would have confounded the strong univariate relationship with rurality. Similar associations between rurality and notification rates of both STEC and cryptosporidiosis in New Zealand have been observed by Thorburn [62], reporting higher rates in rural areas.

Exposures to farming environments were reported as risk factors for sporadic STEC infections in case–control studies conducted in England [63], North America [11], Germany [8], and Argentina [21]. A prospective case–control study by Locking *et al*. [64] identified contact, or likely contact, with animal manure as a strong risk factor for sporadic STEC O157 infection in Scotland, while a retrospective case–control study by Voetsch *et al*. [65] observed direct or indirect contact with cattle manure as a leading source of sporadic STEC O157 infections in North America. We also identified contact with animal manure as a significant risk factor in addition to cattle being present in the meshblock in which the case resided. Both variables were associated with the largest population attributable fractions and, when combined with the spatial analysis and the strong correlation between the presence of cattle, and particularly beef cattle and STEC cases at the meshblock level, it indicates that contact with cattle faeces is the major exposure pathway for infection in New Zealand.

'Travelling to areas of New Zealand with interrupted or no main water supply’ and 'Contact with recreational waters’ emerged as significant environmental risk factors for sporadic STEC infections; activities which increasingly occur in the summer period. Previous studies conducted in Finland [66] and North America [20,67] reported an association between gastrointestinal illnesses, including STEC, after exposure to recreational waters during summer. Such an association could also explain some of the observed seasonality of STEC cases as discussed above. In addition, an estimated 14% of New Zealand’s population is not served by community drinking-water supplies [68] but retrieves drinking water from private springs and bores, streams and creeks, or roof runoffs. This risk applies particularly to residents of rural areas. Considering the large ruminant livestock population in New Zealand, ground water and particularly surface water in rural areas are potentially contaminated with ruminant faeces containing STEC.

We found no evidence to suggest that sporadic STEC cases in New Zealand were associated with exposure to STEC-contaminated food products, while 'Drinking refrigerated fruit juice from supermarket’, 'Eating raw vegetables’ and 'Eating seafood’ were negatively correlated with disease. An inverse effect of fruit and vegetables has also been reported in previous case–control studies conducted in Australia [69] and Scotland [64] and merits further investigation. The association seems biologically plausible compared to other food products, as they are associated with health benefits such as antimicrobial properties against human pathogens in berries [70] and sweet potato leaves [71]. An alternative explanation might be the association between fruit and vegetable consumption and the participants’ choice of healthy eating. This apparent protective effect might also be caused by recall bias, as discussed under *sources of bias*.

### Molecular epidemiology of *E. coli* O157:H7

The molecular analysis of PFGE profiles from human *E. coli* O157:H7 isolates revealed three distinctive clusters of genotypes, each represented by a specific SBI type. SBI types are defined based on the insertion site of the Stx-associated bacteriophage and the presence or absence of *stx* genes in the bacterial genome, which encode for the Shiga toxin proteins. *stx*2c is a subtype of the *stx*2 gene and characteristic for isolates of SBI type 5. The observed clustering was significantly associated with 'Island of residence’, indicating that SBI type 5 was more prevalent in the South Island, whereas SBI types 1 and 3 were more common in the North Island. This distinct geographical difference in genotype distribution was also observed in a recent molecular study including 28 bovine and 209 human *E. coli* O157:H7 isolates originating from both islands of New Zealand [72]. The distinct between-island distributions of genotypes found among bovine and human isolates indicate localised transmission between cattle and humans. SBI type 5 accounted for 20.6% of human isolates in that study, which is much greater than its frequency in other international studies [27,73]. This is consistent with a limited historical introduction of this strain into New Zealand and subsequent evolution.

A significant relationship between SBI types and age categories of cases was observed, in particular between SBI type 5 and 0–4 year-old children. It can be hypothesised, if this genotype possesses host-adapted characteristics to affect specifically the immature gastrointestinal tract of children, or whether the observed association is due to SBI type 5 being a more persistent environmental contaminant to which very young children are more likely to be exposed than adults. Recent studies investigating differential virulence of STEC O157 strains have suggested that STEC O157 strains carrying *stx*2c alone are likely to be less pathogenic compared to strains carrying combinations of *stx*2c and *stx*1 as shown in a piglet model [74], or less potent on human kidney cell lines and in mouse models [75].

The molecular variation of PFGE profiles of the isolates was explained by only one explanatory variable: 'Island of residence’, which was consistent with the observed clustering of isolates. Together with significant associations observed between SBI types and both 'Season’ and 'Contact with animal manure’, these findings provide further evidence of an animal/environmental-associated pathway of sporadic STEC infection in New Zealand.

### Sources of bias

The two week window of exposure might have resulted in some recall bias due to difficulty remembering previous exposures. However, this time period was chosen to cover the likely incubation period for STEC (3–12 days) while lists of possible answers facilitated recall of consumed food items, contact with animal species and environmental exposures. Observational studies of this type can also introduce recall bias due to the cases being more likely to recall events than the non-affected controls [76]. This effect could explain the apparent protective association seen for consumption of a range of foods, where recall was less complete amongst controls, as found in a previous New Zealand case–control study of similar populations [59]. In addition, 31% of cases were interviewed differently to the other cases and controls, which might have introduced some systematic differences between them, though such a bias is unlikely to have had an important effect on the findings.

There was evidence of some selection bias in the control population as a result of using random landline dialling for recruitment. Based on the national census data, the older age group of controls was overrepresented compared to the younger age groups. This might be because younger age groups favouring mobile technology over landlines, or their tendency to reside in relatively fewer households with a larger number of individuals. Nonetheless, little bias was observed in the distribution of ethnicities, gender, and rural/urban living among controls, compared to national census data.

The exclusion of potentially eligible cases had a negligible effect on the findings. Only one case probably acquired their infection overseas so was excluded. There were no apparent outbreaks or clusters of concurrent cases observed during the study period, indicating that secondary infections occur only relatively infrequently in New Zealand suggesting that this study was effectively one of sporadic cases.

The number of confirmed cases reported through the disease surveillance system is likely to be an underestimation of the true incidence of human STEC infections in New Zealand. Scallan *et al*. [77] and Tam *et al*. [78], using different approaches, have estimated under-ascertainment fractions of STEC cases in the USA and the UK. Asymptomatic or mild cases are unlikely to present to medical practitioners and not all stool samples received at diagnostic laboratories are routinely tested for *E. coli* O157:H7 and non-O157 STECs in New Zealand. In addition, the majority of diagnostic laboratories test stool samples of STEC cases for *E. coli* O157:H7 only, which could explain the current predominance of STEC O157. Therefore risks presented could be underestimated, or different measures of association could apply compared to findings in this study.

## Conclusions

Our findings strongly indicate that environmental and animal contact, but not food, are important exposure pathways for sporadic cases of human STEC infection in New Zealand. There are strong indications that dairy cattle and beef cattle are the most important sources of STEC and contact with manure from these animals represents an important exposure pathway. Notably, outbreaks of STEC infections are rare in New Zealand and this further suggests that food is not a significant exposure pathway.

## Competing interests

The authors declare that they have no competing interests.

## Authors’ contributions

PJ designed and coordinated the study, performed the screening for *stx*2c of isolates, performed the statistical analysis and drafted the manuscript; ALC helped with the study design and reviewed the manuscript; DMC conceived of the study, participated in its design, and reviewed the manuscript; TEB and SS performed the SBI genotyping of isolates; GFM helped with study design, coordination of the study, and reviewed the manuscript; EL set up the Survey Gizmo and database, and LL participated in coordination of study and data collection; MD performed the PFGE genotyping of isolates; JCM contributed to the statistical analysis; MGB contributed to the study design and reviewing of the manuscript; SH and DJP contributed to the study design; NPF contributed to the study design, statistical analysis, drafting and reviewing of the manuscript. All authors read and approved the final manuscript.

## Pre-publication history

The pre-publication history for this paper can be accessed here:

http://www.biomedcentral.com/1471-2334/13/450/prepub

## Supplementary Material

Additional file 1 Questionnaire. Questions asked in interview of study cases and controls. Click here for file

Additional file 2 **Ruminant livestock densities in New Zealand from 2011.** Densities (animals/km^2^) of (A) dairy cattle, (B) beef cattle, (C) sheep, and (D) deer in New Zealand from 2011. Click here for file

Additional file 3 Results of bivariate logistic regression analysis (adjusted for age categories). Click here for file

Additional file 4 **Multivariate logistic regression model without imputations.** Results showing identified risk factors after deleting 57 of 619 observations (113 cases and 506 controls) due to missing values. 'No exposure/contact’ was chosen as reference level for comparison in each variable (odds ratio = 1.00). Click here for file
