# Supplementary material for: A prospective case–control and molecular epidemiological study of human cases of Shiga toxin-producing Escherichia coli in New Zealand
Source: BMC Infect Dis. 2013 Sep 30;13:450. doi: 10.1186/1471-2334-13-450 (PMC3854066; doi:10.1186/1471-2334-13-450)
Supplement: Additional file 1 — Questionnaire. Questions asked in interview of study cases and controls. [file 1471-2334-13-450-S1.doc]

# VTEC Questionnaire

## Main: Demographics

1) If child: What is the gender of [insert child's name]?

( ) Male

( ) Female

If adult: Interviewer to enter gender of respondent

( ) Male

( ) Female

2a) Would you mind telling me your / [insert child's name] age please?

*Enter age in years. For a child whose age is stated in months, record the age in years at the child’s last birthday. i.e. 1 to 11 months is '0' years of age, 12 – 23 months is ‘1’ year, 24 – 35 months is '2' years*

( ) Age given in years __________ Go to 3 after age is recorded

( ) Refused to answer – Go to 2b

2b) Would you mind telling which one of the following age groups you / [insert child's name] is in?

( ) 0-4 years

( ) 5-9 years

( ) 10-14 years

( ) 15-19 years

( ) 20-29 years

( ) 30-39 years

( ) 40-49 years

( ) 50-59 years

( ) 60-69 years

( ) 70+ years

( ) Don't know (DO NOT READ)

( ) Refused to answer (DO NOT READ)

3) Which of the following ethnic group(s) do you /does [insert child's name] belong to? One or several groups may apply to you.

[ ] New Zealand European

[ ] New Zealand Maori

[ ] Samoan

[ ] Cook Island Maori

[ ] Tongan

[ ] Niuean

[ ] Chinese

[ ] Indian

[ ] Other Asian (specify)________

[ ] Other1 (specify)________

[ ] Other2 (specify)_________

[ ] Refused to answer (DO NOT READ)

If adult

4) What is your occupation?

*If unknown, enter "Unknown". If respondent refuses to provide an answer, enter "Refused to answer".*

____________________________________________

If child

5) Is [insert child's name] a:

( ) Preschooler

( ) Primary or intermediate student

( ) Secondary student

( ) Tertiary student

( ) Refused to answer

6) What is the name of the nearest school to where you / [insert name] live and what town or city is this school in? This could be a primary, intermediate or secondary school. If there are a number of schools nearby, just name one.

*Reasons for wanting to know the nearest school: have a rough idea of where the case lives. If unknown, enter "Unknown". If refused to answer, enter "Refused to answer"*

Name of school: _________________________

Town/city: _________________________

7) Do you / [insert child's name] live at a rural delivery (RD) address?

( ) Yes

( ) No

( ) Unknown

( ) Refused to answer

## Main: Food

8a) Did you / [insert name] drink any treated milk or eat any dairy products made from treated milk in the 2 weeks before getting sick? (By treated milk we mean heated, treated or pasteurised milk; milk or milk products bought in a shop)

( ) Yes – Go to 8b

( ) No – Go to 9a

( ) Don't know – Go to 9a

( ) Refused to answer – Go to 9a

8b) Can you please specify the type, brand and where you had obtained the dairy or dairy products you / [insert child's name] had consumed?

Type: _________________________

Brand: _________________________

Source: _________________________

9a) Did you / [insert child's name] drink any raw milk or eat any products made from raw milk which you bought directly from a farm in the 2 weeks before getting sick? (By raw milk we mean untreated or unpasterurised milk.)

( ) Yes – Go to 9b

( ) No – Go to 10

( ) Don't know – Go to 10

( ) Refused to answer – Go to 10

9b) Can you please specify the type, brand and where you had obtained the raw milk or any products products made from raw milk you / [insert child's name] had consumed?

Type: _________________________

Brand: _________________________

Source: _________________________

10) Did you / [insert child's name] eat any meat or poultry, excluding eggs, in the 2 weeks before getting sick?

( ) Yes – Go to 11a

( ) No – Go to 21

( ) Don't know – Go to 21

( ) Refused to answer – Go to 21

11a) Did you / [insert child's name] eat any beef meat or beef products that needed to be cooked in the 2 weeks before getting sick?

*Examples: Do not read - list of examples is for reference / clarification only*

*steak, beef sausages, eye fillet, roast beef, beef stir fry, beef stew, beef casserole, beef schnitzel, prime beef, beef curry, veal burger, veal fillet, any type of offal from calf/cattle.*

( ) Yes

( ) No

( ) Don't know

( ) Refused to answer

11b) Did you/ [insert child's name] eat any minced beef that needed to be cooked?

( ) Yes

( ) No

( ) Don't know

( ) Refused to answer

If yes to 11a OR 11b,

11c) Did any of this meat appear undercooked on the inside when you / [insert child's name] ate it?

*Undercooked meat: meat products that need to be cooked thoroughly before eating e.g. meat patties, sausages, meat loafs etc. as they are potential sources of E. coli infection*

( ) Yes

( ) No

( ) Don't know

( ) Refused to answer

If yes to 11a OR 11b,

11d) Can you please specify the type, brand and where you had obtained the beef or beef products consumed?

Type: _________________________

Brand: _________________________

Source: _________________________

12a) Did you / [insert child's name] eat any lamb or mutton (meat of adult sheep) or lamb/ mutton products that needed to be cooked in the 2 weeks before getting sick?

*Examples: Do not read - list of examples is for reference / clarification only*

*lamb shanks, lamb sausages, roast lamb, lamb stew, lamb stir fry, lamb chops, lamb casserole, lamb mince, lamb flaps, lamb patties, lamb curry, roast mutton, any type of offal from lamb/sheep.*

( ) Yes – Go to 12b

( ) No – Go to 13a

( ) Don't know – Go to 13a

( ) Refused to answer– Go to 13a

12b) Did any of the lamb or mutton meat appear undercooked on the inside when you/ [insert child's name] ate it?

*Undercooked meat: meat products that need to be cooked thoroughly before eating e.g. meat patties, sausages, meat loafs etc. as they are potential sources of E. coli infection*

( ) Yes

( ) No

( ) Don't know

( ) Refused to answer

12c) Can you please specify the type, brand and where you had obtained the lamb/mutton or lamb/mutton products consumed?

Type: _________________________

Brand: _________________________

Source: _________________________

13a) Did you / [insert child's name] eat any chicken, other poultry (e.g. duck, goose, turkey or ostrich) or poultry products that needed to be cooked in the 2 weeks before getting sick?

*Examples: Do not read - list of examples is for reference / clarification only*

*roast chicken, chicken sausages, chicken breasts, chicken drumsticks, chicken stir fry, chicken curry, chicken nuggets, roast turkey, turkey breasts, roast goose, duck breasts, ostrich, any type of offal from poultry.*

( ) Yes – Go to 13b

( ) No – Go to 14

( ) Don't know – Go to 14

( ) Refused to answer – Go to 14

13b) Can you please specify the type, brand and where you had obtained the chicken/poultry or chicken/poultry products consumed?

Type: _________________________

Brand: _________________________

Source: _________________________

14) Did you / [insert child's name] eat any pork meat or pork products that needed to be cooked in the 2 weeks before getting sick?

( ) Yes

( ) No

( ) Don't know

( ) Refused to answer

15a) Did you / [insert child's name] eat any venison meat or venison products that needed to be cooked in the 2 weeks before getting sick?

*Examples: Do not read - list of examples is for reference / clarification only*

*venison steak, venison sausages, venison fillet, venison burger, venison mince, any type of offal from deer.*

( ) Yes – Go to 15b

( ) No - Go to 16a

( ) Don't know - Go to 16a

( ) Refused to answer - Go to 16a

15b) Did any of the venison meat or venison products appear undercooked on the inside when you/ [insert child's name] ate it?

*Undercooked meat: meat products that need to be cooked thoroughly before eating e.g. meat patties, sausages, meat loafs etc. as they are potential sources of E. coli infection*

( ) Yes

( ) No

( ) Don't know

( ) Refused to answer

16a) Did you / [insert child's name] eat any meat products such as meat from the delicatessen counter or cold cuts of meat that do not need to be cooked in the 2 weeks before getting sick?

*Examples: Do not read - list of examples is for reference / clarification only*

*any type of meat pies, any type of sandwiches containing sliced meat, any type of kebabs containing meat, bacon, ham, luncheon, salami or other cured meats (pastrami, prosciutto, parma ham, pancetta), any type of sliced cold meat, corned beef/silverside, cheerios, frankfurters, saveloys, meat paté = meat paste, Mettwurst.*

( ) Yes – Go to 16b

( ) No – Go to 17a

( ) Don't know – Go to 17a

( ) Refused to answer – Go to 17a

16b) Can you please specify the type, brand and where you had obtained the meat products (processed meats) such as deli meat or cold cuts consumed?

Type: _________________________

Brand: _________________________

Source: _________________________

17a) Did you / [insert child's name] eat any home kill meat, excluding wild game, in the 2 weeks before getting sick?

*Examples: Do not read - list of examples is for reference / clarification only*

*meat of animals raised and slaughtered on farm, such as pork, beef, veal, lamb, mutton, venison, chicken, turkey.*

( ) Yes – Go to 17b

( ) No – Go to 18a

( ) Don't know – Go to 18a

( ) Refused to answer – Go to 18a

17b) Did any of the home kill meat appear undercooked on the inside when you / [insert child's name] ate it?

( ) Yes

( ) No

( ) Don't know

( ) Refused to answer

18a) Did you/ [insert child's name] eat any wild game meat in the 2 weeks before getting sick?

( ) Yes – Go to 18b

( ) No – Go to 20

( ) Don't know– Go to 20

( ) Refused to answer– Go to 20

18b) Did any of the wild game meat appear undercooked on the inside when you / [insert child's name] ate it?

*Undercooked meat: meat products that need to be cooked thoroughly before eating e.g. meat patties, sausages, meat loafs etc. as they are potential sources of E. coli infection*

( ) Yes

( ) No

( ) Don't know

( ) Refused to answer

If Yes to 17a OR 18a

19) Can you please specify the type of and where you had obtained the home kill or wild game meat consumed?

Type: _________________________

Source: _________________________

20) Did you / [insert child's name] eat any offal, from any species of animal, in the 2 weeks before getting sick?

( ) Yes

( ) No

( ) Don't know

( ) Refused to answer

21) Did you/ [insert child's name] handle or touch any raw meat or offal at home or work, including raw meat or offal given to pets, in the 2 weeks before getting sick? By handled, we do not mean you / [insert child's name] ate it.

( ) Yes

( ) No

( ) Don't know

( ) Refused to answer

22) Did you / [insert child's name] eat any fish or shellfish, excluding seafood from a can, in the 2 weeks before getting sick?

( ) Yes

( ) No

( ) Don't know

( ) Refused to answer

23a) Did you / [insert child's name] eat any raw fruit, either as part of a meal or by themselves, in the 2 weeks before getting sick? (this excludes canned fruit)

*Examples: Do not read - list of examples is for reference / clarification only*

*fresh fruit salad, fruit platters, apples, pears/nashi pears, bananas, berries, citrus fruits (mandarins, oranges, grapefruits, tangelos), kiwi fruit, peaches, nectarines, apricots, cherries, strawberries, blueberries, any other berries, pineapple, grapes, plums, watermelon, rockmelon, passion fruit, feijoas.*

( ) Yes – Go to 23b

( ) No – Go to 24a

( ) Don't know – Go to 24a

( ) Refused to answer – Go to 24a

23b) Were any of the fruits you/ [insert child's name] ate home grown, either by yourself or someone else?

( ) Yes - Go to 23c

( ) No – Go to 24a

( ) Don't know – Go to 24a

( ) Refused to answer – Go to 24a

23c) Was animal manure, fertilizer or compost containing animal manure used to grow the home grown fruits?

( ) Yes

( ) No

( ) Don't know

( ) Refused to answer

24a) Did you / [insert child's name] eat any raw (not cooked) vegetables or herbs, for example salad vegetables such as lettuce, carrots, tomatoes, spinach etc, either as part of a meal or by themselves, in the 2 weeks before getting sick?

*Examples: Do not read - list of examples is for reference / clarification only*

*bean sprouts or similar, watercress, lettuce or other salad leaves, silver beet, spinach, fresh herbs, puha, carrots, beetroot, coleslaw, celery, tomatoes, cucumber, courgettes, capsicum, fennel, egg plant, sweet corn, broccoli, cauliflower, spring onions, peas / snow peas or similar, parsley, chives, other herbs, mushrooms.*

( ) Yes – Go to 24b

( ) No – Go to 25a

( ) Don't know – Go to 25a

( ) Refused to answer – Go to 25a

24b) Were any of the vegetables you/ [insert child's name] ate home grown, either by yourself or someone else?

( ) Yes – Go to 24c

( ) No – Go to 25a

( ) Don't know – Go to 25a

( ) Refused to answer – Go to 25a

24c) Was animal manure, fertilizer or compost containing animal manure used to grow the home grown vegetables or herbs?

( ) Yes

( ) No

( ) Don't know

( ) Refused to answer

If Yes to 23b OR 24b

24d) Please specify type of raw fruit / vegetables consumed that were homegrown.

____________________________________________

If Yes to 23a OR 24a

24e) Can you please specify the type, brand and where you had obtained the any raw fruit or vegetables consumed that were not homegrown?

Type: _________________________

Brand: _________________________

Source: _________________________

25a) Did you/ [insert child's name] drink any fruit or vegetable juice purchased from the refrigerated section of a supermarket or shop in the 2 weeks before getting sick?

*Note: Does* ***NOT*** *include fruit/vegetable juice bought from dry goods shelves (shelf stable juice)*

( ) Yes – Go to 25b

( ) No – Go to 26a

( ) Don't know – Go to 26a

( ) Refused to answer – Go to 26a

25b) Can you please specify type and brand of fruit / vegetable juice bought from the refrigerated section of the supermarket or shop?

*Note: Does* ***NOT*** *include fruit/vegetable juice bought from dry goods shelves (shelf stable juice)*

Type: _________________________

Brand: _________________________

26a) Did you / [insert child's name] drink any fruit or vegetable juice prepared fresh from a café/ restaurant/ juice bar or at home in the 2 weeks before getting sick? This includes grasses.

( ) Yes – Go to 26b

( ) No - Go to 27a

( ) Don't know - Go to 27a

( ) Refused to answer - Go to 27a

26b) Please specify type and source of juice prepared fresh

Type: _________________________

Source: _________________________

## Main: Dining Locations

27a) Did you/ [insert child's name] eat any food prepared and/or cooked outside of your home kitchen in the 2 weeks before getting sick?

*Examples: Do not read – list of examples is for reference / clarification only*

*National hamburger chain (McDonalds, Burger King, KFC, Wendy’s), national pizza chain (Hells Pizza, Dominos, Pizza Hut), Subway, other takeaway outlets, fish and chips shop, bakery, café/restaurant/pub, food bought at petrol stations, other location with professional caterers (e.g. wedding), other location without professional caterers (e.g. BBQ at friends, birthday/family party, work party, social event at sports club, Xmas/New Year’s party, school function, school/sports camp, hangi, umu), at an agricultural show (e.g. Field days)/petting zoo/wildlife park, BBQ at home.*

( ) Yes – Go to 27b

( ) No – Go to 28a

( ) Don't know – Go to 28a

( ) Refused to answer – Go to 28a

27b) Did any of the meat you / [insert child's name] ate, prepared and/ or cooked outside of your home kitchen, appear undercooked on the inside?

*Undercooked meat: meat products that need to be cooked thoroughly before eating e.g. meat patties, sausages, meat loafs etc. as they are potential sources of E. coli infection*

( ) Yes

( ) No

( ) Don't know

( ) Refused to answer

## Main: Drinking Water

28a) Which of the following is the main source of water supply to your/ [insert child's name] home/ house?

( ) Usual town supply - Go to 29a

( ) Private bore / spring water - Go to 28b

( ) Tanker truck water - Go to 28b

( ) Roof / rain water - Go to 28b

( ) Creek / stream water - Go to 28b

( ) Don't know - Go to 29a

28b) If not town supply, is the water treated or filtered?

( ) Yes – Go to 28c

( ) No - Go to 29a

( ) Don't know - Go to 29a

( ) Refused to answer - Go to 29a

28c) How is the water treated or filtered?

*S*elect as many as apply.

|  | **Yes** | **No** | **Don't know** |
| --- | --- | --- | --- |
| Chlorinated | ( ) | ( ) | ( ) |
| Boiled | ( ) | ( ) | ( ) |
| Filtered | ( ) | ( ) | ( ) |
| Treated with UV | ( ) | ( ) | ( ) |
| Don't know | ( ) | ( ) | ( ) |

29a) If adult

Which of the following is the main source of water supply at your workplace / [insert child’s name’s] school?

( ) Usual town supply - Go to 30

( ) Private bore / spring water - Go to 29b

( ) Tanker truck water - Go to 29b

( ) Roof / rain water - Go to 29b

( ) Creek / stream water - Go to 29b

( ) Don't know - Go to 30

If child

Which of the following is the main source of water supply at [insert child's name's] school?

( ) Usual town supply - Go to 30

( ) Private bore / spring water - Go to 29b

( ) Tanker truck water - Go to 29b

( ) Roof / rain water - Go to 29b

( ) Creek / stream water - Go to 29b

( ) Don't know - Go to 30

29b) If not town supply, is the water treated or filtered?

( ) Yes – Go to 29c

( ) No – Go to 30

( ) Don't know – Go to 30

( ) Refused to answer – Go to 30

29c) How is the water treated or filtered?

*S*elect as many as apply.

|  | **Yes** | **No** | **Don't know** |
| --- | --- | --- | --- |
| Chlorinated | ( ) | ( ) | ( ) |
| Boiled | ( ) | ( ) | ( ) |
| Filtered | ( ) | ( ) | ( ) |
| Treated with UV | ( ) | ( ) | ( ) |
| Don't know | ( ) | ( ) | ( ) |

30) Were there any problems or changes in the regular water supply to your home/ house or workplace or [insert child's name]'s school in the 2 weeks before getting sick?
*For example, changes due to natural disasters (e.g. earthquakes or flooding), technical issues and therefore forced to use other water sources such as untreated water on a farm, bore water, tanker/ truck water, roof/ rain water, untreated creek/ stream water.*

( ) Yes

( ) No

( ) Don't know

( ) Refused to answer

31a) In the 2 weeks before getting sick, did you / [insert child's name] drink any water from supplies that you would not normally drink from? We mean outside of your home, work, school, etc.

( ) Yes – Go to 31b

( ) No – Go to 32a

( ) Don't know – Go to 32a

( ) Refused to answer – Go to 32a

31b) Specify the type(s) of water supply.

*Select all that apply*

[ ] Town supply

[ ] Private bore / spring water

[ ] Tanker truck water

[ ] Roof / rain water

[ ] Creek / stream water

[ ] Don't know

## Main: Contact with Recreational Waters

32a) Did you/ [insert child's name] swim in a public swimming pool in the 2 weeks before getting sick?

( ) Yes – Go to 32b

( ) No - Go to 33a

( ) Don't know - Go to 33a

( ) Refused to answer - Go to 33a

32b) Can you please specify the name and location of the public swimming pool(s)?

____________________________________________

33a) Did you/ [insert child's name] swim in any other private swimming pools in the 2 weeks before getting sick?

( ) Yes – Go to 33b

( ) No - Go to 34a

( ) Don't know - Go to 34a

( ) Refused to answer - Go to 34a

33b) Would you mind telling me the name and/or location of the private pool(s)?

____________________________________________

34a) Did you/ [insert child's name] use a spa or paddling pool in the 2 weeks before getting sick?

( ) Yes – Go to 34b

( ) No - Go to 35a

( ) Don't know - Go to 35a

( ) Refused to answer - Go to 35a

34b) Was that a spa pool or a paddling pool, or both? If spa pool, please specify name and/or location of pool(s).

*Select all that apply.*

[ ] Paddling pool

[ ] Refused to answer

[ ] Spa pool (specify name and location) _______________________________________

35a) Did you/[insert child's name] take part in recreational activities in fresh water such as in a stream, river, lake, dam or pond in the 2 weeks before getting sick? This does not include salt or sea water.

( ) Yes – Go to 35b

( ) No - Go to 36a

( ) Don't know - Go to 36a

( ) Refused to answer - Go to 36a

35b) Please specify location of recreational activities in fresh water such as stream, river, lake, etc.

____________________________________________

36a) Did you / [insert child's name] have any contact with sewage, stagnant water or flood waters in the 2 weeks before getting sick?

( ) Yes – Go to 36b

( ) No - Go to 37a

( ) Don't know - Go to 37a

( ) Refused to answer - Go to 37a

36b) Which of the following did you / [insert child's name] have contact with?

*Select all that apply*

[ ] Sewage

[ ] Stagnant waters

[ ] Flood waters

[ ] Refused to answer (DO NOT READ)

## Main: Hunting Activities

37a) Did you / [insert child's name] go hunting in the 2 weeks before getting sick?

( ) Yes – Go to 37b

( ) No – Go to 38a

( ) Don't know – Go to 38a

( ) Refused to answer – Go to 38a

37b) Did you / [insert child's name] handle any of the following animals or birds as a result of the hunting trip?

*Select as many as apply*

[ ] Pig

[ ] Boar

[ ] Deer

[ ] Goat

[ ] Thar

[ ] Chamois

[ ] Possum

[ ] Rabbit

[ ] Duck

[ ] Pheasant

[ ] Other (specify) __________________

[ ] Refused to answer (DO NOT READ)

## Main: Animal Contact

38a) Did you / [insert child's name] have any physical contact with your own or other people's household pets in the 2 weeks before getting sick? If no contact, please just say so.

( ) Yes – Go to 38b

( ) No – Go to 39

( ) Don't know – Go to 39

( ) Refused to answer – Go to 39

38b) What household pets did you / [insert child's name] have physical contact with in the 2 weeks before getting sick?

*Select as many as apply*

[ ] Dog

[ ] Cat

[ ] Rabbits

[ ] Guinea pig / hamster / gerbil

[ ] Mouse / rat

[ ] Aviary birds

[ ] Chickens / other poultry

[ ] Horse / pony / donkey

[ ] Fish tank or plants

[ ] Turtle

[ ] Other reptile (e.g. snake)

[ ] Other (specify) __________________

[ ] Refused to answer (DO NOT READ)

39) Do you / [insert child's name] live on a farm / lifestyle block with animals?

( ) Yes

( ) No

( ) Don't know

( ) Refused to answer

40a) Did you / [insert child's name] have any physical contact with animals other than household pets in the 2 weeks before getting sick? If no contact, please just say so.

( ) Yes – Go to 40b

( ) No – Go to 41

( ) Don't know – Go to 41

( ) Refused to answer – Go to 41

40b) Which of the following animals did you / [insert child's name] have contact with in the 2 weeks before getting sick?

*Select as many as apply*

[ ] Horse / pony / donkey

[ ] Chickens / poultry

[ ] Cattle (including dairy cows / calves)

[ ] Pigs / piglets

[ ] Sheep / lamb

[ ] Deer

[ ] Llamas / alpacas

[ ] Working dog

[ ] Farm cat

[ ] Aviary birds

[ ] Other animals in a zoo or wildlife park

[ ] Other (specify) __________________

[ ] Don't know

[ ] Refused to answer (DO NOT READ)

If Yes to 38a OR 40a,

41) What kind of contact did you / [insert child's name] have with the animals, was it:

*Select as many as apply*

[ ] General farm work with animals

[ ] Job related (e.g. slaughter house worker, veterinarian, animal trainer, milker, shepherd, stock agent)

[ ] Riding / walking / exercising

[ ] Feeding

[ ] Petting / stroking

[ ] Cleaning / bathing the animal(s), grooming

[ ] Cleaning cages / kennels / stables etc

[ ] Picking up faeces / manure

[ ] Giving medication

[ ] Other (specify) __________________

[ ] Don't know

[ ] Refused to answer (DO NOT READ)

42a) Did you / [insert child's name] have any intentional or unintentional contact with animal manure / faeces or compost containing animal manure / faeces in the 2 weeks before getting sick?

( ) Yes – Go to 42b

( ) No – Go to 43a

( ) Don't know – Go to 43a

( ) Refused to answer – Go to 43a

42b) What type of animal manure / faeces was this?

*Read list - select all that apply*

[ ] Bird droppings

[ ] Cattle / calf

[ ] Sheep / lamb

[ ] Horse / pony / donkey

[ ] Chicken / poultry

[ ] Pigs

[ ] Dog

[ ] Cat

[ ] Rabbit

[ ] Mice / rat

[ ] Guinea pig / hamster / gerbil

[ ] Compost from garden centre

[ ] Fertilizer or compost made from animal manure

[ ] Don't know

[ ] Refused to answer (DO NOT READ)

43a) Did anyone else in your household other than you / [insert child's name] have contact with animals other than household pets in the 2 weeks before getting sick?

( ) Yes – Go to 43b

( ) No – Go to 44

( ) Don't know – Go to 44

( ) Refused to answer – Go to 44

43b) What kind of contact did this household member have with the animals, was it:

*Select as many as apply*

[ ] General farm work with animals

[ ] Job related (e.g. slaughter house worker, veterinarian, animal trainer, milker, shepherd, stock agent)

[ ] Feeding

[ ] Petting / stroking

[ ] Cleaning / bathing the animal(s), grooming

[ ] Cleaning cages / kennels / stables etc

[ ] Picking up faeces / manure

[ ] Giving medication

[ ] Other (specify) __________________

[ ] Don't know

[ ] Refused to answer (DO NOT READ)

## Main: Human Contact

44) Did you / [insert child's name] visit a school, pre-school or childcare facility in the 2 weeks before getting sick?

( ) Yes

( ) No

( ) Don't know

( ) Refused to answer

45) Did you / [insert child's name] have any contact with children in nappies in the 2 weeks before getting sick?

( ) Yes

( ) No

( ) Don't know

( ) Refused to answer

46a) Did you / [insert child's name] have any contact with a **person with vomiting or any symptoms of diarrhoea or (gastrointestinal) disease** in the 2 weeks before getting sick? For example, at a rest or nursing home, at a care/health centre in town, at the doctor/clinic, at a hospital.

( ) Yes – Go to 46b

( ) No – Go to 47a

( ) Don't know – Go to 47a

( ) Refused to answer – Go to 47a

46b)  Could you please tell me the nature of contact you had with them and when they had gotten sick?

Nature of contact: ____________________________________________

Date of onset of illness in other case (dd/mm/yyyy): _____________________

47a) Did you / [insert child's name] attend any social functions in the 2 weeks before getting sick?

( ) Yes – Go to 47b

( ) No – Go to 48

( ) Don't know – Go to 48

( ) Refused to answer – Go to 48

47b) Can you please specify details of the function(s)?

***DO NOT*** *record any names (or identifiable information) here. Specify type, size, and location of function (e.g. children's party at home of case - 30 people attended)*

____________________________________________

____________________________________________

____________________________________________

## Main: Travel

48) In the 2 weeks before getting sick, did you / [insert child's name] visit a town/area in New Zealand apart from where you usually live and work, where there was no main water supply or an interruption in the usual water supply (e.g. Christchurch / recent flooding)

( ) Yes

( ) No

( ) Don't know

( ) Refused to answer

49a) In the 2 weeks before getting sick, did you / [insert child's name] travel within New Zealand?

( ) Yes

( ) No

( ) Don't know

( ) Refused to answer

49b) Please specify where in New Zealand you / [insert child's name] had travelled to?

____________________________________________

## Main: Antibiotic/Antacid Use

Instructions/additional notes for interviewer are in grey. Do not read out any information provided in grey. Questions/details for the respondent are in black.

50) Did you / [insert child's name] take any antibiotics in the 2 weeks before getting sick?

( ) Yes

( ) No

( ) Don't know

( ) Refused to answer

51) Did you / [insert child's name] take any antacids/pills to reduce stomach acid in the 2 weeks before getting sick?

( ) Yes

( ) No

( ) Don't know

( ) Refused to answer
