# Supplementary figures and images for: A prospective case–control and molecular epidemiological study of human cases of Shiga toxin-producing Escherichia coli in New Zealand
Source: BMC Infect Dis. 2013 Sep 30;13:450. doi: 10.1186/1471-2334-13-450 (PMC3854066; doi:10.1186/1471-2334-13-450)

## Slide 1
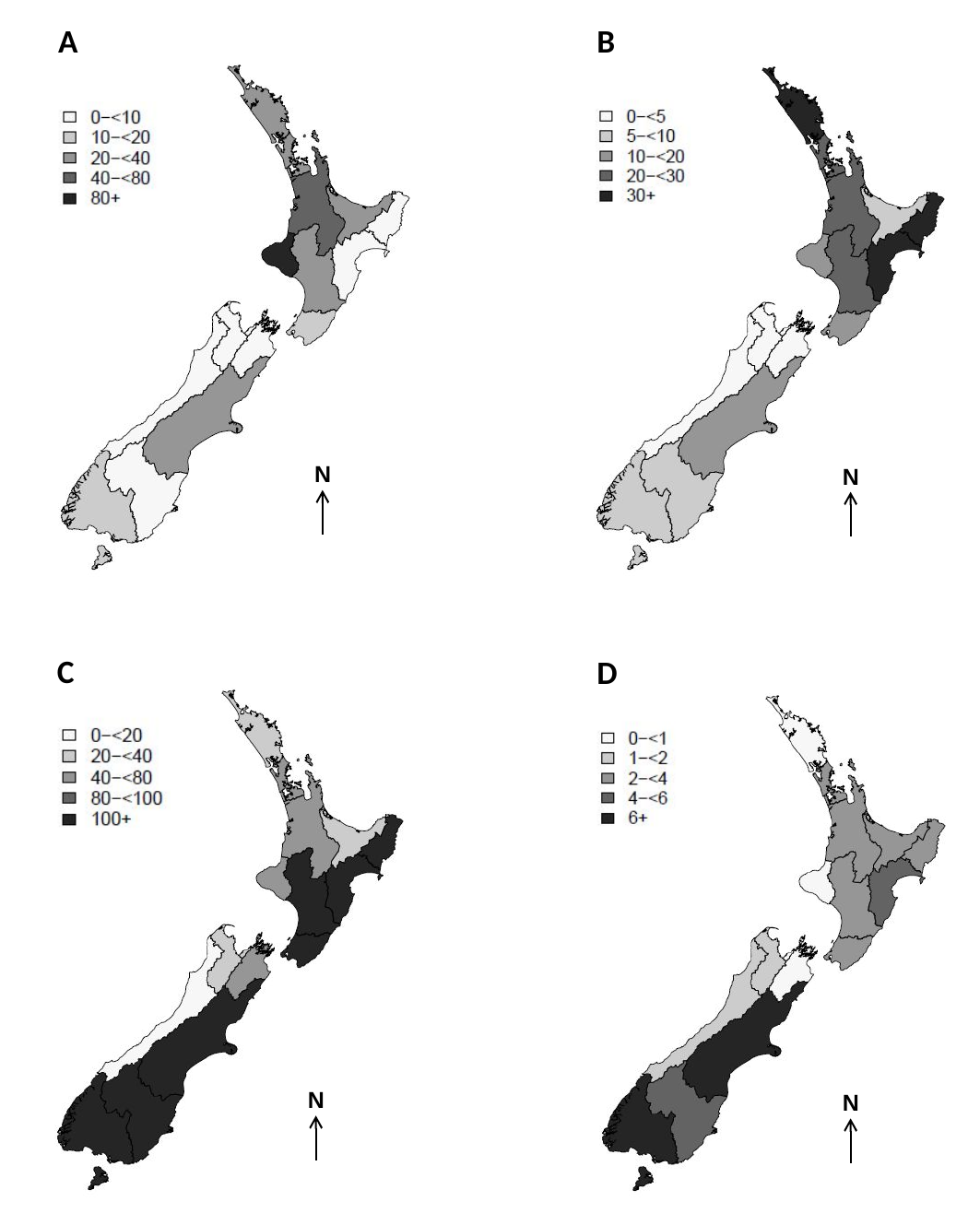

A
B
N
N
C
D
N
N

Supplement: Additional file 2 — Ruminant livestock densities in New Zealand from 2011. Densities (animals/km2) of (A) dairy cattle, (B) beef cattle, (C) sheep, and (D) deer in New Zealand from 2011. [file 1471-2334-13-450-S2.pptx]
