# Supplementary material for: A prospective case–control and molecular epidemiological study of human cases of Shiga toxin-producing Escherichia coli in New Zealand
Source: BMC Infect Dis. 2013 Sep 30;13:450. doi: 10.1186/1471-2334-13-450 (PMC3854066; doi:10.1186/1471-2334-13-450)
Supplement: Additional file 3 — Results of bivariate logistic regression analysis (adjusted for age categories). [file 1471-2334-13-450-S3.docx]

| Variable | | Cases | Controls | Coefficient (SE)^a^ | Odds ratio  (95% CI)^b^ | Wald test  *p*-value |
| --- | --- | --- | --- | --- | --- | --- |
| *Demographic data* | |  |  |  |  |  |
| Gender | |  |  |  |  |  |
|  | Male | 59 | 217 | Ref | - | - |
|  | Female | 54 | 289 | -0.30 (0.21) | 0.74 (0.49 - 1.13) | 0.165 |
| Season | |  |  |  |  | 0.021* |
|  | Spring (Sep - Nov) | 21 | 128 | Ref | - | - |
|  | Summer (Dec - Feb) | 28 | 126 | 0.33 (0.32) | 1.39 (0.75 - 2.60) | 0.294 |
|  | Autumn (Mar - May) | 38 | 126 | 0.59 (0.30) | 1.80 (1.00 - 3.25) | 0.051 |
|  | Winter (Jun - Aug) | 26 | 126 | 0.24 (0.32) | 1.27 (0.68 - 2.38) | 0.458 |
| Ethnicity | |  |  |  |  | 0.005* |
|  | NZ European | 98 | 415 | Ref | - | - |
|  | Maori | 8 | 23 | 0.27 (0.43) | 1.30 (0.56 - 3.04) | 0.538 |
|  | Others | 7 | 68 | -0.84 (0.42) | 0.43 (0.19 - 0.97) | 0.042 |
| Occupation | |  |  |  |  | 0.004* |
|  | Baby/Preschooler | 52 | 206 | Ref | - | - |
|  | Student (primary school to Uni) | 17 | 62 | 0.77 (1.37) | 2.16 (0.15 - 31.69) | 0.574 |
|  | Professional/Management | 13 | 83 | 3.58 (1.80) | 35.90 (1.07 - 1207.32) | 0.046 |
|  | Trade/Agricultural/Unemployed | 18 | 77 | 3.67 (1.75) | 39.07 (1.28 - 1195.34) | 0.036 |
|  | Retired | 12 | 78 | 3.56 (1.80) | 35.26 (1.04 - 1190.56) | 0.047 |
|  | NA | 1 | - |  |  |  |
| Location within New Zealand | |  |  |  |  | <0.001* |
|  | Upper half of North Island | 62 | 243 | Ref | - | - |
|  | Lower half of North Island | 12 | 127 | -0.98 (0.34) | 0.37 (0.19 - 0.72) | 0.003 |
|  | Upper half of South Island | 24 | 86 | 0.06(0.27) | 1.06 (0.62 - 1.81) | 0.829 |
|  | Lower half of South Island | 15 | 50 | 0.17 (0.33) | 1.19 (0.62 - 2.26) | 0.606 |
| Living in rural area | |  |  |  |  |  |
|  | No | 71 | 401 | Ref | - | - |
|  | Yes | 42 | 102 | 0.82 (0.23) | 2.26 (1.45 - 3.52) | <0.001 |
|  | NA | - | 3 |  |  |  |
|  |  |  |  |  |  |  |
| *Food associated risk factors* | |  |  |  |  |  |
| Drinking treated milk | |  |  |  |  |  |
|  | No | 12 | 60 | Ref | - | - |
|  | Yes | 101 | 446 | 0.16 (0.34) | 1.17 (0.60 - 2.29) | 0.638 |
| Drinking raw milk | |  |  |  |  |  |
|  | No | 108 | 490 | Ref | - | - |
|  | Yes | 5 | 16 | 0.30 (0.53) | 1.35 (0.48 - 3.79) | 0.574 |
| Eating meat | |  |  |  |  |  |
|  | No | 11 | 48 | Ref | - | - |
|  | Yes | 101 | 458 | 0.02 (0.36) | 1.02 (0.50 - 2.06) | 0.961 |
|  | NA | 1 | - |  |  |  |
| Handling raw offal | |  |  |  |  |  |
|  | No | 95 | 350 | Ref | - | - |
|  | Yes | 16 | 156 | -0.91 (0.31) | 0.40 (0.22 - 0.74) | 0.003 |
|  | NA | 2 | - |  |  |  |
| Eating seafood | |  |  |  |  |  |
|  | No | 67 | 215 | Ref | - | - |
|  | Yes | 41 | 291 | -0.72 (0.22) | 0.48 (0.31 - 0.75) | 0.001 |
|  | NA | 5 | - |  |  |  |

| Variable | | Cases | Controls | Coefficient (SE)^a^ | Odds ratio (95% CI)^b^ | Wald test *p*-value |
| --- | --- | --- | --- | --- | --- | --- |
| Eating raw fruit | |  |  |  |  |  |
|  | No | 14 | 43 | Ref | - | - |
|  | Yes | 99 | 462 | -0.41 (0.33) | 0.67 (0.35 - 1.28) | 0.223 |
|  | NA | - | 1 |  |  |  |
| Eating raw vegetables | |  |  |  |  |  |
|  | No | 37 | 76 | Ref | - | - |
|  | Yes | 73 | 430 | -1.07 (0.25) | 0.34 (0.21 - 0.56) | <0.001 |
|  | NA | 3 | - |  |  |  |
| Drinking refrigerated fruit juice from supermarket | |  |  |  |  |  |
|  | No | 94 | 280 | Ref | - | - |
|  | Yes | 18 | 222 | -1.49 (0.28) | 0.23 (0.13 - 0.39) | <0.001 |
|  | NA | 1 | 4 |  |  |  |
| Drinking fresh fruit juice from café | |  |  |  |  |  |
|  | No | 101 | 405 | Ref | - | - |
|  | Yes | 8 | 97 | -1.20 (0.39) | 0.30 (0.14 - 0.65) | 0.002 |
|  | NA | 4 | 4 |  |  |  |
| Dining outside home | |  |  |  |  |  |
|  | No | 40 | 100 | Ref | - | - |
|  | Yes | 70 | 405 | -0.88 (0.23) | 0.41 (0.26 - 0.65) | <0.001 |
|  | NA | 3 | 1 |  |  |  |
|  |  |  |  |  |  |  |
| *Water associated risk factors* | |  |  |  |  |  |
| Water supply to home from | |  |  |  |  |  |
|  | Town supply/tanker/roof run off | 89 | 468 | Ref | - | - |
|  | Private bore/spring/creek/ stream | 22 | 35 | 1.16 (0.30) | 3.19 (1.77 - 5.72) | <0.001 |
|  | NA | 2 | 3 |  |  |  |
| Visiting areas of NZ without main water supply or recently interrupted main water supplies | |  |  |  |  |  |
|  | No | 96 | 473 | Ref | - | - |
|  | Yes | 15 | 28 | 0.94 (0.35) | 2.56 (1.29 - 5.05) | 0.007 |
|  | NA | 2 | 5 |  |  |  |
| Swimming in public pool | |  |  |  |  |  |
|  | No | 94 | 407 | Ref | - | - |
|  | Yes | 19 | 98 | -0.51 (0.29) | 0.60 (0.34 - 1.07) | 0.083 |
|  | NA | - | 1 |  |  |  |
| Swimming in private pool | |  |  |  |  |  |
|  | No | 105 | 460 | Ref | - | - |
|  | Yes | 8 | 46 | -0.52 (0.41) | 0.60 (0.27 - 1.33) | 0.209 |
| Swimming in spa pool | |  |  |  |  |  |
|  | No | 106 | 442 | Ref | - | - |
|  | Yes | 7 | 63 | -0.97 (0.42) | 0.38 (0.17 - 0.87) | 0.021 |
|  | NA | - | 1 |  |  |  |
| Recreational activities in fresh water | | |  |  |  |  |
|  | No | 97 | 474 | Ref | - | - |
|  | Yes | 15 | 32 | 0.69 (0.34) | 1.99 (1.02 - 3.86) | 0.043 |
|  | NA | 1 | - |  |  |  |
| Contact with sewage/standing water/flood waters | |  |  |  |  |  |
|  | No | 105 | 486 | Ref | - | - |
|  | Yes | 6 | 19 | 0.39 (0.48) | 1.47 (0.57 - 3.8) | 0.426 |
|  | NA | 2 | 1 |  |  |  |

| Variable | | Cases | Controls | Coefficient (SE)^a^ | Odds ratio (95% CI)^b^ | | Wald test *p*-value |
| --- | --- | --- | --- | --- | --- | --- | --- |
| *Animal associated risk factors* | |  |  |  |  |  | |
| Beef livestock present in meshblock | |  |  |  |  |  | |
|  | No | 74 | 422 | Ref | - | - | |
|  | Yes | 39 | 84 | 0.96 (0.23) | 2.61 (1.65 - 4.13) | <0.001 | |
| Dairy livestock present in meshblock | |  |  |  |  |  | |
|  | No | 94 | 477 | Ref | - | - | |
|  | Yes | 19 | 29 | 1.18 (0.32) | 3.26 (1.74 - 6.13) | <0.001 | |
| Sheep livestock present in meshblock | |  |  |  |  |  | |
|  | No | 82 | 434 | Ref | - | - | |
|  | Yes | 31 | 72 | 0.78 (0.25) | 2.19 (1.35 - 3.57) | 0.002 | |
| Deer livestock present in meshblock | |  |  |  |  |  | |
|  | No | 105 | 488 | Ref | - | - | |
|  | Yes | 8 | 18 | 0.72 (0.44) | 2.05 (0.86 - 4.88) | 0.106 | |
| Living on farm/lifestyle block with animals | |  |  |  |  |  | |
|  | No | 72 | 423 | Ref | - | - | |
|  | Yes | 41 | 83 | 1.01 (0.23) | 2.74 (1.74 - 4.31) | <0.001 | |
| Contact with household pets | |  |  |  |  |  | |
|  | No | 28 | 140 | Ref | - | - | |
|  | Yes | 82 | 366 | 0.03 (0.24) | 1.03 (0.64 - 1.67) | 0.897 | |
|  | NA | 3 | - |  |  |  | |
| Contact with animals other than household pets | |  |  |  |  |  | |
|  | No | 74 | 415 | Ref | - | - | |
|  | Yes | 37 | 90 | 0.76 (0.24) | 2.14 (1.35 - 3.39) | 0.001 | |
|  | NA | 2 | 1 |  |  |  | |
| Exposure to animal manure or compost containing animal manure | |  |  |  |  |  | |
|  | No | 66 | 413 | Ref | - | - | |
|  | Yes | 34 | 86 | 0.96 (0.25) | 2.60 (1.61 - 4.22) | <0.001 | |
|  | NA | 13 | 7 |  |  |  | |
| Hunting activities | |  |  |  |  |  | |
|  | No | 112 | 500 | Ref | - | - | |
|  | Yes | 1 | 6 | -0.58 (1.10) | 0.56 (0.06 - 4.86) | 0.600 | |
| Other household member having contact with animals other than household pets | |  |  |  |  |  | |
|  | No | 71 | 407 | Ref | - | - | |
|  | Yes | 41 | 95 | 0.84 (0.23) | 2.31 (1.47 - 3.63) | <0.001 | |
|  | NA | 1 | 4 |  |  |  | |
|  |  |  |  |  |  |  | |
| *Human associated risk factors* | |  |  |  |  |  | |
| Visiting childcare/pre-school/school facilities | |  |  |  |  |  | |
|  | No | 72 | 250 | Ref | - | - | |
|  | Yes | 41 | 256 | -1.07 (0.25) | 0.34 (0.21 - 0.56) | <0.001 | |
| Contact with children in nappies | |  |  |  |  |  | |
|  | No | 66 | 253 | Ref | - | - | |
|  | Yes | 41 | 249 | -0.75 (0.26) | 0.47 (0.29 - 0.78) | 0.004 | |
|  | NA | 6 | 4 |  |  |  | |
| Contact with person vomiting or having gastrointestinal disease | |  |  |  |  |  | |
|  | No | 88 | 414 | Ref | - | - | |
|  | Yes | 15 | 76 | -0.08 (0.31) | 0.92 (0.50 - 1.68) | 0.788 | |
|  | NA | 10 | 16 |  |  |  | |
| Variable | | Cases | Controls | Coefficient (SE)^a^ | Odds ratio (95% CI)^b^ | | Wald test *p*-value |
| *Host associated risk factors* | |  |  |  |  |  | |
| Taking antibiotics | |  |  |  |  |  | |
|  | No | 99 | 456 | Ref | - | - | |
|  | Yes | 11 | 48 | 0.11 (0.36) | 1.11 (0.56 - 2.24) | 0.760 | |
|  | NA | 3 | 2 |  |  |  | |
| Taking antacids | |  |  |  |  |  | |
|  | No | 106 | 448 | Ref | - | - | |
|  | Yes | 4 | 57 | -0.94 (0.55) | 0.39 (0.13 - 1.15) | 0.087 | |
|  | NA | 3 | 1 |  |  |  | |
| ^a^ SE = Standard error.  ^b^ CI = 95% confidence interval.  * *p*-value of variable as whole using Likelihood ratio test.  Ref = reference level for comparison. NA = observations with either 'unknown' or missing values. | | | | | | | |
