# Supplementary material for: A prospective case–control and molecular epidemiological study of human cases of Shiga toxin-producing Escherichia coli in New Zealand
Source: BMC Infect Dis. 2013 Sep 30;13:450. doi: 10.1186/1471-2334-13-450 (PMC3854066; doi:10.1186/1471-2334-13-450)
Supplement: Additional file 4 — Multivariate logistic regression model without imputations. Results showing identified risk factors after deleting 57 of 619 observations (113 cases and 506 controls) due to missing values. 'No exposure/contact’ was chosen as reference level for comparison in each variable (odds ratio = 1.00). [file 1471-2334-13-450-S4.docx]

| Variable | | Coefficient (SE)^a^ | Odds ratio (95% CI)^b^ | Wald test *p*-value |
| --- | --- | --- | --- | --- |
| Other household member having contact with animals other than household pets^c^ | |  |  |  |
|  | for 0-4 year-old | 2.30 (0.55) | 10.01 (3.38 - 29.71) | <0.001 |
|  | for 5-19 year-old | -0.15 (0.74) | 0.86 (0.20 - 3.64) | 0.838 |
|  | for >19 year-old | 0.07 (0.58) | 1.08 (0.35 - 3.35) | 0.899 |
| Cattle livestock present in meshblock | | 1.12 (0.35) | 3.07 (1.53 - 6.15) | 0.002 |
| Exposure to animal manure or compost containing animal manure | | 0.82 (0.36) | 2.27 (1.11 - 4.63) | 0.025 |
| Recreational activities in fresh water | | 1.41 (0.50) | 4.10 (1.55 - 10.85) | 0.004 |
| Visiting areas of New Zealand without main water supply or recently interrupted main water supplies | | 1.42 (0.47) | 4.15 (1.64 - 10.49) | 0.003 |
| Water supply to home from  private bore/ spring/ creek/ or stream | | 0.61 (0.47) | 1.85 (0.73 - 4.68) | 0.195 |
| Handling raw offal | | -1.26 (0.42) | 0.29 (0.12 - 0.65) | 0.003 |
| Eating seafood | | -0.58 (0.32) | 0.56 (0.30 - 1.06) | 0.074 |
| Eating raw vegetables | | -0.58 (0.41) | 0.56 (0.25 - 1.25) | 0.156 |
| Drinking refrigerated fruit juice from supermarket | | -1.35 (0.39) | 0.26 (0.12 - 0.56) | 0.001 |
| Dining outside home | | -0.62 (0.34) | 0.54 (0.28 - 1.06) | 0.073 |
| Contact with children in nappies | | -0.21 (0.37) | 0.81 (0.39 - 1.68) | 0.576 |
| Visiting childcare/pre-school/school facilities | | -1.68 (0.41) | 0.19 (0.08 - 0.42) | <0.001 |
| Taking antacids | | -1.03 (0.67) | 0.36 (0.10 - 1.33) | 0.124 |
| Likelihood ratio test = 152.94 (df = 18, *p*<0.001)  ^a^ SE = Standard error  ^b^ CI = 95% confidence interval  ^c^ This variable was modelled using a multiplicative interaction term comprising the variables ‘Other household member having contact with animals other than household pets’ and ‘Age’.  It can be interpreted as follows: a child 0-4 years of age is at significantly higher risk of being an STEC case, if another household member had contact with animals other than household pets, compared to a child of the same age without this risk factor. | | | | |
